# Subthalamic deep brain stimulation in Parkinson's disease with *SNCA* mutations: Based on the follow‐up to 10 years

**DOI:** 10.1002/brb3.2503

**Published:** 2022-01-18

**Authors:** Jinyoung Youn, Genko Oyama, Nobutaka Hattori, Yasushi Shimo, Tomi Kuusimäki, Valtteri Kaasinen, Angelo Antonini, Dongyeop Kim, Jung‐Il Lee, Kyung Rae Cho, Jin Whan Cho

**Affiliations:** ^1^ Departments of Neurology Samsung Medical Center Sungkyunkwan University School of Medicine Seoul Republic of Korea; ^2^ Neuroscience Center Samsung Medical Center Seoul Republic of Korea; ^3^ Department of Neurology Juntendo University School of Medicine Tokyo Japan; ^4^ Neurocenter Turku University Hospital Turku Finland; ^5^ Clinical Neurosciences, Faculty of Medicine University of Turku Turku Finland; ^6^ Parkinson and Movement Disorders Unit, Department of Neurosciences University of Padua Padua Italy; ^7^ Departments of Neurosurgery Samsung Medical Center Sungkyunkwan University School of Medicine Seoul Republic of Korea; ^8^ Departments of Neurosurgery Konkuk University Medical Center Seoul Republic of Korea

**Keywords:** alpha‐synuclein, deep brain stimulation, Parkinson, *SNCA*, subthalamus

## Abstract

**Backgrounds:**

Although the short‐term efficacy of bilateral subthalamic deep brain stimulation (DBS) has been reported in a limited number of Parkinson's disease (PD) patients with *SNCA* mutations, there are no data for long‐term outcome.

**Methods:**

This multicenter retrospective study investigated previously reported PD patients with *SNCA* mutations, implanted with bilateral subthalamic DBS. We compared demographic and clinical data at baseline and last follow‐up. Clinical data of motor and nonmotor symptoms and motor fluctuation were collected up to 10 years from DBS surgery.

**Results:**

Among four subjects, three had *SNCA* duplication and one had c.158C.A (p.A53E) mutation. The mean post‐implantation follow‐up duration was 5.4 ± 3.7 years. All patients with *SNCA* duplication showed favorable outcome, although one died from breast cancer 1.5 years after DBS. The patient with the missense mutation became wheelchair‐bound due to progressed axial, cognitive and psychiatric symptoms after 3.5 years from DBS despite the benefit on motor fluctuation.

**Conclusion:**

Based on findings in our small cohort, subthalamic DBS could be beneficial for motor fluctuation in PD patients with *SNCA* mutations, especially those with *SNCA* duplication, and cognitive and psychiatric symptoms are important for the long‐term outcome of subthalamic DBS.

## Abstract 2

1. This study assesses the long‐term outcome of bilateral subthalamic deep brain stimulation (DBS) in patients with Parkinson's disease (PD) related with SNCA mutations.
2. Based on our results, we suggest bilateral subthalamic DBS could be effective treatment option in PD with SNCA mutations if the candidates are well‐selected.
3. Cognitive, psychiatric symptoms and axial motor symptoms are important for the long‐term outcome of subthalamic DBS in PD with SNCA mutation.

## 1 INTRODUCTION

Various α‐synuclein gene (*SNCA*) mutations including missense mutations and multiplications were identified in autosomal dominant Parkinson's disease (PD) (Lill, 2016). PD patients with *SNCA* mutations demonstrate earlier age at onset and commonly had motor fluctuations, even compared to those with other autosomal dominant genetic PD (Wittke et al., 2018). Therefore, deep brain stimulation (DBS) could be considered in PD with *SNCA* mutations (Book et al., 2018; Wittke et al., 2018). One previous review demonstrated most monogenic PD patients showed at least short‐term benefit from subthalamic nucleus (STN) DBS (Kuusimaki et al., 2019), but the long‐term efficacy is still unclear. Further, considering high prevalence of cognitive or psychiatric problems in PD with *SNCA* mutations (Book et al., 2018; Wittke et al., 2018), long‐term outcome of STN DBS is important when we take DBS into account as a possible treatment option in PD patients from *SNCA* mutations. Therefore, we investigated long‐term outcome of bilateral STN DBS in PD patients with *SNCA* mutation (missense mutations and multiplications) by a worldwide multicenter retrospective and cross‐sectional study.

## 2 METHODS

This study was approved by the Institutional Review Board of Samsung Medical Center and involved hospitals. Although this study is not a systemic review, we used the previous guideline for a systemic review to find reported STN DBS cases with *SNCA* mutations (Page et al., 2021), and then we collected the long‐term outcome by contacting the authors of each reported case. Literature searches were conducted in PubMed search from inception on 1 February 2019 with keywords “*SNCA* or α‐synuclein gene” and “deep brain stimulation or DBS.” The initial search identified 31 articles with English language. Exclusion criteria was (1) studies with PD patients with nongenetic causes or monogenic etiology but not *SNCA* mutation, (2) studies with DBS on other targets, (3) review or other studies without original data, or (4) animal study. Initial screening was independently done by 2 researchers (J Youn and JW Cho), and disagreements were resolved after discussion. Fourteen duplicated studies and 14 nonrelevant studies (studies with PD patients with genetic mutations other than *SNCA* mutations or without subthalamic DBS [*n* = 12] and animal studies [*n* = 2]) were excluded. Finally, three patients from three case reports were selected we contacted three centers with previous relevant studies to participate in this retrospective study (Antonini et al., 2012; Martikainen et al., 2015; Shimo et al., 2014). Additionally, we also included 1 PD patient with *SNCA* duplication with bilateral STN DBS at Samsung Medical Center, Seoul, Korea.

All the data at the baseline (pre‐DBS), and the last follow‐up after DBS (long‐term outcome) were collected from February 2019 to February 2020. Demographic data including age, age at the onset of PD, age at DBS, family history and follow‐up duration, and clinical outcome about motor and nonmotor symptoms and fluctuation were collected using the standardized data collection form in all enrolled subjects. The clinical outcome from DBS was assessed based on the three categories; (1) motor symptoms using Unified Parkinson's disease rating scale (UPDRS) part 3 and Hoehn and Yahr stage, (2) nonmotor symptoms with mini‐mental status exam (MMSE) and Beck's depression inventory, and (3) fluctuation/dyskinesia with the item #32 and #39 of UPDRS part 4. We added other clinical scales used at each center.

## 3 RESULTS

### 3.1 Phenotypes and genotypes of enrolled subjects

Of totally four PD patients with *SNCA* mutations, duplication was identified in three (case #1–3) (Antonini et al., 2012; Shimo et al., 2014), and missense mutation (c.158C.A [p.A53E]) in one (case #4) (Martikainen et al., 2015), and all the data were illustrated in Table 1. The mean age and disease duration at DBS were 44.3 ± 2.8 years and 5.5 ± 0.6 years. Mean UPDRS part 3 score was 10.3 ± 0.5 (ON status) and 29.3 ± 2.1 (OFF status). Levodopa equivalent dose (LED) was 938.8 ± 261.3 mg/day and mean frequency for medication as 7.8 ± 2.4 per day. No patients demonstrated dementia and mean MMSE score was 29.3 ± 1.0 before DBS. There were no surgical complications, while device‐related complication was reported in case #4. In case #4, DBS was removed 1 year after surgery because of infection and reimplanted 6 months after removal.

**TABLE 1:** Baseline demographic and clinical data of enrolled subjects with SNCA mutation

	Case #1		Case #2		Case #3		Case #4	
Identified mutation	duplication		duplication		duplication		c.158C.A (p.A53E)	
Age at onset	41		35		37		42	
Age at DBS	46		41		43		47	
Gender	female		male		female		female	
Family history	+		+		–		+	
Follow‐up duration after DBS (years)	1.5		10		6.5		3.5	

Evaluation	Baseline	Last visit	Baseline	Last visit	Baseline	Last visit	Baseline	Last visit
Motor								
UPDRS part 3^†^	10	12	11	16	11	7	10	56
HY stage	1	2	3	2	1	2		5
Other motor symptoms	off dystonia				off FOG	minimal off FOG		dysarthria, sialorrhea
Non‐motor symptoms								
MMSE	30	24	29	23	30	30	28	18
BDI	6	10	5	8	1	1	11	30
Other non‐motor symptoms	FAB: 14.7, ICD, Constipation, Urinary urgency, Fatigue	FAB 13.5, Constipation, Urinary urgency, Fatigue	FAB: 11, hallucination, constipation	FAB: 18, hallucination	BAI: 13, sleep disturbance	BAI: 4, mild hallucination	mild hallucination, anxiety, pain	PLC, hallucination, anxiety, OH, EDS
Fluctuation								
Duration of wearing off^‡§^	3	1	2	1	2	1		
Duration of dyskinesia^‡§^	2	1	1	0	1	0		
Other information for fluctuation							AIMS: 28	AIMS: 1
LED (mg/day)	1105	400	925	600	1150	370	575	475
Number of doses per day (/day)	7	3	6	3	8	2	11	5

Abbreviations: BAI, Beck's anxiety inventory; BDI, Beck's depression inventory; DBS, deep brain stimulation; EDS, excessive daytime sleepiness, other information for fluctuation; FAB, frontal assessment battery; HY, Hoehn and Yahr; ICD, impulse control disorder; LED, levodopa equivalent doses; MMSE, mini‐mental status exam; OH, orthostatic hypotension; PLC, pathologic laughing and crying; UPDRS, unified Parkinson's disease rating scale.

^†^ UPDRS part 3 was evaluated during ‘ON’ status for baseline, and ‘medication‐ON and stimulation‐ON’ status for last visit.

^‡§^ Duration was scored based on UPDRS part 4 item #32 and #39: 0: none, 1: 1%–25% of day, 2: 26%–50% of day, 3: 51%–75% of day, 4: 75%–100% of day.

### 3.2 Outcome of bilateral subthalamic DBS

Two subjects with *SNCA* duplication (case #2 and #3) were followed up to 6.5 and 10 years from DBS and revealed excellent motor outcome from bilateral STN DBS (Table 1). Nonmotor symptoms were also stable in these subjects. Another patient with *SNCA* duplication (case #1) died from breast cancer 1.5 years after DBS, but her parkinsonism was well‐controlled till her death.

In the subject with missense mutation (case #4), disease itself progressed with levodopa‐nonresponsive or ‐induced symptoms such as depression, perceptional problem, and cognitive decline. However, motor benefit from STN DBS was still evident, considering she still took PD medications less frequently with less dyskinesia compared to the baseline.

## 4 DISCUSSION

This is the first study to investigate the outcome of STN DBS up to 10 years in the PD patients with *SNCA* mutations. Despite small number of patients, our results suggest a good outcome in the three cases with duplications whereas outcome in the patient with the missense mutation was not robust because of axial symptoms and cognitive and psychiatric problems.

For the motor fluctuation/dyskinesia, STN DBS showed favorable outcome in all cases regardless of genotypes. Fluctuation/dyskinesia was dramatically controlled in two patients with *SNCA* duplication (case #2 and #3) 10 and 6.5 years after DBS, and in another (case #1) until she died 1.5 years after DBS. Although one with missense mutation (case #4) had unfavorable outcome, subthalamic stimulation was still effective for motor fluctuation and dyskinesia till the last follow‐up.

Despite the sustained benefits for fluctuation/dyskinesia, axial motor symptoms, dementia, and psychiatric symptoms were important factors to decide the outcome from STN DBS in PD patients with SNCA mutations in our study. In particular, cognitive and psychiatric symptoms are common in *SNCA* mutations (Book et al., 2018; Wittke et al., 2018). Previous meta‐analysis of *SNCA* multiplication revealed that dementia was noted in half of this cohort although clinical presentations varied throughout (Book et al., 2018). In our study, two subjects with favorable long‐term outcome (case #2 and #3) demonstrated relatively stable cognition and hallucination during entire follow‐up up to 10 years from DBS, and anxiety even improved in one of two subjects (case #3). On the other hands, cognitive and psychiatric symptoms were deteriorated as well as axial motor symptoms in a patient (case #4) who showed unfavorable outcome from STN DBS.

In spite of motor benefit from STN DBS, the different outcomes in cognitive function among monogenic PDs were already reported (Artusi et al., 2019). Similarly, based on the different outcome between the PD patients with *SNCA* duplication and missense mutation, genotype could affect the outcome of STN DBS. Among *SNCA* missense mutation, duplication, and triplication, *SNCA* triplications had the earliest onset, and duplications had the latest onset. Considering PD with *SNCA* triplication shows much more aggressive disease progression involving both motor and nonmotor symptoms including cognitive and psychiatric symptoms, DBS itself may not be considered in the patients with *SNCA* triplication and this can be the reason why there is no case report with DBS case with *SNCA* triplication. On the other hand, *SNCA* duplication could present with relatively slow progressive PD compared to triplication or missense mutation. *SNCA* duplication was also detected in sporadic PD patients (Ahn et al., 2008), and the phenotype may resemble idiopathic PD, including late age‐of‐onset, slow progression, and less dementia (Chartier‐Harlin et al., 2004). Therefore, these could be the reasons why DBS was mostly performed in PD patients with *SNCA* duplication (Antonini et al., 2012; Perandones et al., 2015; Shimo et al., 2014). However, the progression could be rapid even in PD with *SNCA* duplication. Indeed, the mother of case #1 revealed rapid progression, and showed full dementia 7 years after disease onset (Antonini et al., 2012). Therefore, even in PD patients with *SNCA* duplication, it is important to choose those with stable cognitive and psychiatric symptoms as candidate for STN DBS.

Another issue is about the target for DBS in PD patients with *SNCA* mutation. There is only one report with pallidal stimulation (Perandones et al., 2015). Considering *SNCA* mutation is associated with psychiatric symptoms or cognitive decline (Book et al., 2018; Wittke et al., 2018), pallidal stimulation could be safer option than subthalamic stimulation. However, PD patients with *SNCA* mutation are relatively young, thus there is more need to reduce PD medication. Moreover, the reduction of medication dose is also important to improve perceptional problem. In our study, hallucination was seen in three of four subjects (case #2–4) but deteriorated only in one subject with missense mutation (case #4) after DBS. Additionally, even with STN DBS, many studies reported preserved or improved cognition or psychiatric symptoms (Boel et al., 2016). Therefore, we suggest STN could be preferred target than globus pallidus interna if the candidates with *SCNA* mutations had stable cognitive and psychiatric symptoms.

Our study has some limitations. Our results were based on small number of cases, and it is difficult to generalize to all PD patients with *SNCA* mutation or even to all patients with *SNCA* duplication. Additionally, we recruited PD patients with *SNCA* mutation, who already had performed bilateral STN DBS. This may result in a selection bias that rapidly progressive patients with *SNCA* mutation should not be even considered as a candidate for DBS. Lastly, this is a retrospective study, thus we could not use unified protocol for evaluation of efficacy. However, we tried our best to illustrate all the scales and evaluations as possible.

In conclusion, based on findings in our small cohort, STN DBS is beneficial for motor fluctuation, especially those with *SNCA* duplication, and cognitive and psychiatric symptoms were important factors for the outcome in PD patients with *SNCA* mutations.

## CONFLICT OF INTEREST

J. Youn declared speaker's honoraria from SK chemicals, and Boston Scientific. G. Oyama has been funded by grants from the Japan Society for the Promotion of Science, Grant‐in‐Aid for Scientific Research; He received speaker honoraria from Medtronic, Boston Scientific, Otsuka Pharmaceutical Co. Ltd., Sumitomo Dainippon Pharma Co. Ltd., Eisai Co., Ltd., Takeda Pharmaceutical Company LTD., Kyowa Hakko Kirin Co. Ltd., and AbbVie, Inc. N. Hattori received speaker honoraria from AbbVie GK, EA Pharma, Eisai Co., Ltd., Otsuka Pharmaceutical Co., Ltd., Ono Pharmaceutical Co., Ltd., OHARA Pharmaceutical Co.,Ltd, Kyowa Kirin Co., Ltd., Senju Pharmaceutical Co.,Ltd., Sumitomo Dainippon Pharma Co., Ltd., Takeda Pharma Co., Ltd., Medtronic, Inc., Novartis Pharma K.K. Y. Shimo was funded by grants from the Japan Society for the Promotion of Science, Grant‐in‐Aid for Scientific Research; He received speaker honoraria from Medtronic, Boston Scientific, Otsuka Pharmaceutical, Takeda Pharmaceutical CO, Sumitomo Dainippon Pharma, Novartis Pharma, MSD, FP Pharmaceutical Corporation, Kyowa Hakko Kirin, and AbbVie, Inc. V. Kaasinen received speaker's honoraria from Abbvie, and Nordic Infucare AB; He serves as advisory board for AbbVie, and Nordic Infucare AB, and consultant for Nordic Infucare AB. A. Antonini has received compensation for consultancy and speaker related activities from UCB, Boehringer Ingelheim, Britannia, AbbVie, Zambon, Bial, Neuroderm, Theravance Biopharma, Roche; he receives research support from Chiesi Pharmaceuticals, Lundbeck, Horizon 2020 ‐ Grant 825785, Horizon2020 Grant 101016902, Ministry of Education University and Research (MIUR) Grant ARS01_01081, Cariparo Foundation; He serves as consultant for Boehringer–Ingelheim for legal cases on pathological gambling. J.I. Lee received research support from Medtronic and Boston Scientific. J.W. Cho, T. Kuusimäki, D. Kim, K.R. Cho have no conflict of interest to declare.

## AUTHOR CONTRIBUTION

**JY**: Research project conception, organization, execution; statistical analysis design, execution; manuscript preparation writing of the first draft, review and critique. **GO**: Research project conception, execution; statistical analysis execution, review and critique; manuscript preparation writing of the first draft, review and critique. **NH**: Statistical analysis execution, review and critique; manuscript review and critique. **YS**: Research project organization, execution; statistical analysis review and critique; manuscript preparation review and critique. **TK**: Research project conception, organization, execution, statistical analysis execution, manuscript preparation review and critique. **VK**: Research project organization, execution, statistical analysis review and critique, manuscript preparation review and critique. **AA**: Research project organization, execution, statistical analysis review and critique, manuscript preparation review and critique. **JIL**: Research project organization, execution, statistical analysis review and critique, manuscript preparation review and critique. **DK**: Research project organization, statistical analysis execution; manuscript preparation review and critique. **KRC**: Research project Organization, execution, statistical analysis review and critique; manuscript preparation review and critique

### PEER REVIEW

The peer review history for this article is available at https://publons.com/publon/10.1002/brb3.2503

## Data Availability

All the data used in this study was included in the manuscript.
